# Island biology and morphological divergence of the Skyros wall lizard *Podarcis gaigeae*: a combined role for local selection and genetic drift on color morph frequency divergence?

**DOI:** 10.1186/1471-2148-10-269

**Published:** 2010-09-02

**Authors:** Anna Runemark, Bengt Hansson, Panayiotis Pafilis, Efstratios D Valakos, Erik I Svensson

**Affiliations:** 1 Section for Animal Ecology, Ecology Building, Lund University, SE-223 62 Lund, Sweden; 2 School of Natural Resources and Environment, University of Michigan, Dana Building, 430 East University, Ann Arbor, MI 48109-1115, USA; 3 Modern Greek Program, Department of Classical Studies, 2160 Angell Hall, University of Michigan, 435 S. State, Ann Arbor, MI 48109-1115, USA; 4 Section of Animal and Human Physiology, Department of Biology, University of Athens, Panepistimiopolis, 157 84 Athens, Greece

## Abstract

**Background:**

Patterns of spatial variation in discrete phenotypic traits can be used to draw inferences about the adaptive significance of traits and evolutionary processes, especially when compared to patterns of neutral genetic variation. Population divergence in adaptive traits such as color morphs can be influenced by both local ecology and stochastic factors such as genetic drift or founder events. Here, we use quantitative color measurements of males and females of Skyros wall lizard, *Podarcis gaigeae*, to demonstrate that this species is polymorphic with respect to throat color, and the morphs form discrete phenotypic clusters with limited overlap between categories. We use divergence in throat color morph frequencies and compare that to neutral genetic variation to infer the evolutionary processes acting on islet- and mainland populations.

**Results:**

Geographically close islet- and mainland populations of the Skyros wall lizard exhibit strong divergence in throat color morph frequencies. Population variation in throat color morph frequencies between islets was higher than that between mainland populations, and the effective population sizes on the islets were small (N_e_:s < 100). Population divergence (F_ST_) for throat color morph frequencies fell within the neutral F_ST_-distribution estimated from microsatellite markers, and genetic drift could thus not be rejected as an explanation for the pattern. Moreover, for both comparisons among mainland-mainland population pairs and between mainland-islet population pairs, morph frequency divergence was significantly correlated with neutral divergence, further pointing to some role for genetic drift in divergence also at the phenotypic level of throat color morphs.

**Conclusions:**

Genetic drift could not be rejected as an explanation for the pattern of population divergence in morph frequencies. In spite of an expected stabilising selection, throat color frequencies diverged in the islet populations. These results suggest that there is an interaction between selection and genetic drift causing divergence even at a phenotypic level in these small, subdivided populations.

## Background

The role of genetic drift in population divergence and speciation has been a much debated topic in evolutionary biology [1-5]. Early theoretical models of speciation, so-called "peak-shift models", suggested that genetic drift must be a strong force if a population was to move from one adaptive peak to another, and hence cross a valley with lower fitness [5,6]. These early models generated much controversy and it was later argued that these proposed scenarios were unlikely to be important in population divergence and speciation [1,2]. More recent theoretical models have generated renewed interest for the possibility of genetic drift in speciation. These new models suggest that genetic drift can operate when there exist neutral adaptive ridges in multivariate "holey adaptive landscapes" [3]. Several recent empirical and theoretical studies suggest possible interactions between stochastic factors and selection during evolutionary divergence. For instance, genetic drift can influence population divergence in adaptive phenotypic traits during a short and transient period if selection is temporally relaxed [7-10]. Genetic drift has also been suggested to promote speciation, especially when drift interacts with sexual selection [11]. However, empirical evidence for these scenarios is still rather limited, especially the possible interactions between genetic drift and selection. More generally, various forms of stochasticity have been ignored in most evolutionary studies, and it has been pointed out that genetic drift can also take place in large populations [12].

It is an empirical challenge to argue for a role for genetic drift at the level of phenotypes, since most biologists, for good reasons, believe that phenotypic evolution is mainly influenced by the deterministic forces of natural or sexual selection [13,14]. One possible approach might be to study whether the population distribution of a heritable phenotypic character differs from the neutral expectation or not [7,15,16] . Neutral genetic markers can be used to estimate the effective population sizes (N_e_), and from such data, the expected probability of fixation of neutral and selected alleles can be calculated. The strength of genetic drift is inversely proportional to N_e _and hence drift is a more important force in small populations [17]. If it can be demonstrated that N_e _is small, the potential for genetic drift would increase, both at the phenotypic and at the molecular levels. It is important to emphasize that stochastic factors such as drift, historical contingencies and the initial genetic makeup of populations, could interact with deterministic factors like selection [18-20]. Thus, the critical issue here is not whether genetic drift and stochastic factors *solely *influence population divergence of phenotypic traits, but rather if, and to what extent, stochastic factors might interact with deterministic factors such as selection during evolutionary diversification [21].

Discrete and visible color polymorphisms are excellent phenotypic model systems for the study of evolutionary processes in natural settings [22-24]. Past studies of such polymorphisms have addressed some central issues in population genetics and evolutionary biology, ranging from the question of how genetic variation is maintained in natural populations to speciation processes [23-25]. Color is often also genetically and phenotypically correlated to suites of other morphological, physiological and behavioral traits, e.g. testosterone levels, aggression and disease resistance [26-29]. When the genetic basis of such color polymorphisms is known, the theoretical and analytical framework of population genetics can be used to infer the selective causes behind the spatial variation and the temporal dynamics of morph frequencies [22,25]. Moreover, such studies of the morphs can be used in combination with population estimates of neutral or nearly neutral molecular genetic markers [7,15]. Such color polymorphisms sometimes seem to have a relatively simple genetic basis (one or a few loci) [22]; as appears to be the case for the side-blotched lizard (*Uta stansburiana*) where throat color segregates as a single locus (Mendelian) character [30-32] and the Australian Painted dragon (*Ctenophorus pictus*) where a similar simple inheritance pattern might operate [33]. In the brown Anoline lizard (*Anolis sagrei*), a two-locus inheritance system with epistatic interactions seems to be involved in the determination of back pattern morphs [34].

Although a genetic background for the throat color polymorphism in lizards is often assumed [30-34], no cross-breeding studies have been done to investigate whether it would be governed by a single locus or multiple loci (but see [33]), and the gene(s) involved has not been mapped at the molecular level. It has also been argued that throat color in *Lacerta vivipara *[35] is a continuous character, although [36] argues that it is a discrete, mendelian trait. An additional role for phenotypic plasticity, where the same genetic background has different outcomes in different environments [37], can not be excluded. The high heritabilities estimated from some studies [31] indicate that the role of phenotypic plasticity is small in comparison to the genetic component. Although one can certainly raise caveats for some of these previous studies, and a simple genetic basis can be questioned in some cases, it is quite clear that color polymorphisms in lizards are often heritable, making such traits useful model systems in studies of the relative role of genetic drift vs. selection.

Mechanisms that might contribute to maintain color polymorphisms in populations include negative frequency-dependent male-male competition [26,38], negative frequency-dependent male mating harassment [25], female preferences for unfamiliar mates [39-41], selection in spatially and temporally heterogeneous habitats [42] and sexual selection in combination with microhabitat-specific selection [43]. If a selection pressure connected to a well-defined environmental factor operates on such color morphs, the morph frequencies are expected to change in a parallel fashion across similar ecological environments. In contrast, if genetic drift or other stochastic factors are the predominant evolutionary forces acting on the color morphs, then no such parallelism between morph frequencies and environments are expected. On the other hand, if the color morphs are maintained by negative frequency-dependent selection of a similar kind and magnitude across all populations, then at evolutionary equilibrium, morph frequency divergence should be significantly lower than neutral genetic divergence [15]. In contrast, if directional selection acts on the color morphs in a heterogeneous ecological environment, one would expect more pronounced population divergence in morph frequencies than expected for the neutral genetic variation [44,45]. Finally, under temporally varying selection, genetic drift might partly influence morph frequencies during periods of relaxed selection [7,9,23], which could result in a pattern of morph frequency divergence that would not be discernible from the neutral expectation.

Polymorphic taxa can also shed light on speciation processes; such morphs can serve as starting material for the formation of new species, a process that might be caused by social selection in sympatry [46,47]. Two or more morphs can diverge in different directions already in sympatry, and accelerated evolutionary divergence might subsequently follow if one of these initial morphs later becomes fixed by selection or by stochastic factors in a population [23,43,47].

**Figure 1:**
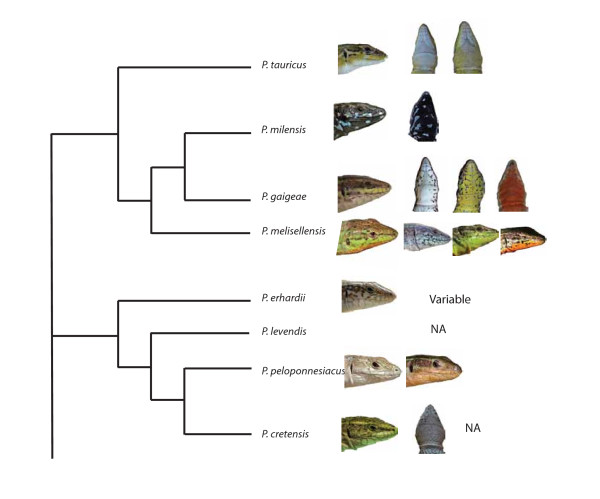
**Throat color variation in the Balkan *Podarcis*-clade**. Examples of some of the intra- and interspecific variation in throat color which is found in the Balkan Peninsula group of *Podarcis *shown on a pruned phylogenetic tree, whose topology was obtained from recent molecular studies [51,90]. Note especially the presence of intraspecific throat color polymorphisms in at least two species (*P. gaigeae *and *P. mellisilensis*) and the entire black throat of the island endemic *P. milensis *on Milos.

In many lizard species, including the European *Podarcis*-clade, color polymorphisms are widespread and multiple throat color morphs coexist within populations [27,48,49] (Fig. 1). Here we present the results of a study of the endemic Skyros wall lizard (*Podarcis gaigeae*) where we study the frequency distribution of throat color morphs in different populations to infer the nature of the selective processes that are acting on the throat color morphs in mainland- and islet populations of this species. This lizard occurs on the island of Skyros in Greece and also on many of the small islets located along the coast of Skyros [48]. The lizard populations on these islets also exhibit substantial morphological inter-islet differentiation, and there are some examples of island gigantism [50]. This biogeographical setting of a large "mainland" area (Skyros) surrounded by smaller islets isolated from the mainland by a few hundred meters of sea is an ideal and classic setting to address issues about the role of stochasticity versus local selection in the divergence of phenotypic traits. In this study, we used molecular (microsatellite) markers to estimate neutral divergence in islet- and mainland populations, and compared the neutral genetic divergence to the divergence in throat color morph frequency. Based on these comparisons we discuss the relative role of genetic drift on morph frequencies in these different geographical settings.

## Methods

### Study species, geographical setting and natural history of islands

**Figure 2:**
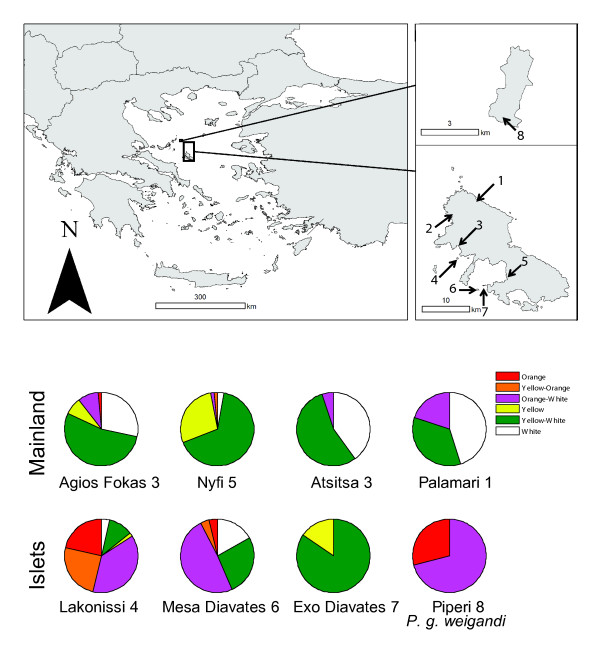
**Distribution of *P. gaigeae *and frequencies of the different throat color morphs in the populations**. (A) Field sampling localities for *P. gaigeae *individuals. Map A shows Greece. The boxes, which are enlarged in map B and C, outline the island of Piperi and the island of Skyros, respectively. Field sampling sites are numbered from one to eight (1: Palamari, 2: Atsitsa, 3: Agios Fokas, 4: Lakonissi, 5: Nyfi, 6: Mesa Diavates, 7: Exo Diavates, 8: the island of Piperi). (B) Throat color morph frequencies among the seven populations of *P. g. gaigeae *and *P. g. weigandi *on Piperi. The upper row refers to populations from the main island of Skyros and the lower to islets close to Skyros and to the island of Piperi.

The Skyros wall lizard *P. gaigeae *is a small bodied insectivorous lacertiid lizard. It is endemic to the Greek island Skyros and its surrounding archipelago [51] with the exception of the subspecies, *P. g. weigandi *which is found on the island of Piperi [48]. For comparative reasons, this subspecies was also included in this study to compare morph frequencies within *P. g. gaigeae *between populations on Skyros, the islets surrounding Skyros and the geographically isolated *P. g. weigandi*. The fragmentation of the landmass which led to the formation of the archipelago of Skyros, and hence the separation from Piperi, had occurred by the early Eemian period [52,53]. Today Piperi is situated 40 kilometers north of Skyros (Fig. 2A). Populations of *P. g. gaigeae *on small islets close to the coast of Skyros show pronounced morphological differentiation, including at least two cases of island gigantism (A. Runemark and E. I. Svensson, unpublished data). Predation rates on islands are often reduced [54] which is also the case for the islets in the Skyros archipelago [50]. The islets close to the coast of Skyros are known for their highly differentiated floral composition and substantial inter-islet variation, and the patterns show some signals of community drift [55].

### Field work and sample collection

Lizards were caught at eight different geographic localities, hereafter referred to as "populations". *P. g. gaigeae *was caught at four different "mainland" localities on the island of Skyros and on three islets close to the coast of Skyros. Lizards of the subspecies *P. g. weigandi *were caught on the island of Piperi (Fig. 2A). Two of the mainland populations and two islet populations were sampled during two consecutive years to ensure that there were no large fluctuations in throat color morph frequency between years. A small part of the tail was collected and immediately preserved it in 99% ethanol for subsequent DNA extraction. All captured animals were photographed with a DMC FX01 Panasonic color camera in an optical isolated box using the built-in xenon flash. Constant illumination was verified with a white background reference. Specular reflections were avoided with two crossed polarization filters. The sex of the lizards was determined through inspection of the femoral pores which are more developed in males than in females [48]. All animals were released after measurement and samples had been taken and sampling was approved by the Hellenic Herpetological Society and the National Sea Park of Alonissos North Sporades.

### Quantitative color analysis of morphs

The lizards were visually classified to one of six clearly discernible throat color categories ("morphs": orange (O), yellow-orange (YO), orange-white (OW), yellow (Y), yellow-white (YW) or white (W)). All these six phenotypes exhibited either one or a combination of any two of the three colors (orange, yellow and white) present in the system, from inspection of the photographic images. No instances of all three colors on the throat of the same lizard were found. To evaluate the reliability of this discrete visual classification system, another observer (E. I. Svensson) independently re-classified a total of 40 lizards that had previously been classified by A. Runemark. In all but two cases (38 out of 40), the independent classifications were identical. This suggests that these color morphs are easily distinguishable with little ambiguity between different observers, possibly reflecting the discrete nature of these morphs.

To further quantify the discreteness of these color morphs, we analyzed the throat area from the images of the individual lizards and used the data in a more quantitative analysis. A total of 351 lizard throats, 320 *P. g. gaigeae *and 31 *P. g. weigandi*, were photographed and visually classified to different color morphs from the images. Due to the variance in intensity, which was related to the imaged scenario geometry (flash distances and surface orientation) rather than the intrinsic optical properties of the lizard, the analysis was pursued with only the relative unit less quantities (S. Svanberg, personal communication). The intensity of the throat images was removed by a transformation of the colorspace from Cartesian to spherical which decreases dimensionality through removing the intensity dimension. Since the aim of the study was to quantify the red green blue (RGB) composition rather than the intensity of the throat colors the subsequent analysis was facilitated by the discarding of the intensity. To preserve the discrete patchy colors of the throat, the chromaticities of individual pixels were summarized in 2 D histogram planes for each sample [56]. The histograms were decomposed using a principal component analysis [57], and the number of principal component planes included in the subsequent analysis was determined by the break point where the difference in the drop between successive eigenvalues is markedly smaller and evens out. Matlab^® ^(MathWorks™) was used for all image analyses.

We subsequently used a discriminant function analysis (DFA) to assess if the visually classified color morphs could be distinguished from each other using the principal component data that we extracted from the images. The software STATISTICA [58] was used for all analyses based on the principal component loadings for each lizard that were extracted from Matlab. We used Spearman rank order correlation to investigate if proportion of orange females was correlated to proportion orange males in each of the populations. We used χ^2^-tests to investigate if the frequencies of the throat color morphs differed among populations and between the habitat groups ("mainland" vs. "islets"). Our starting assumption was a simple (Mendelian) inheritance system: we thus assume that the throat color morph is governed by a single locus with three color alleles, codominant inheritance and hence six visible phenotypes. Based on this assumption, we also tested if the populations deviated from Hardy-Weinberg equilibrium with respect to the throat color morph frequencies, using χ^2^-tests.

Although we have no experimental data (e.g. breeding experiments) on the possible inheritance of throat color in this system, our assumption of a simple genetic basis of color is well-supported by several recent previous studies on color polymorphisms in both lizards and many other animal species (see references in Introduction). We do certainly not claim that there are no environmental effects on color and no role for phenotypic plasticity in these morphs. However, the discrete nature of these color morphs in combination with previous studies on animal coloration makes it highly likely that there is some genetic influence on coloration, perhaps through some major effect gene. Previous studies on color polymorphisms, including studies on birds, fish, lizards and many insect species indicate that variation in color is often heritable, and it is certainly not unusual that color polymorphisms are governed by simple systems of inheritance, e.g. single locus systems with several alternate alleles (see [22] for a recent review). In addition, our analyses do not rely on whether one- or two loci are involved in determining the throat color, but are based on the frequencies of the different alleles, and our results will thus not depend critically on the number of involved loci.

### DNA extraction and microsatellite typing

DNA from all lizard samples was extracted with an ammonium acetate extraction protocol [59]. The DNA was quantified and diluted to 10 ng/μl and used as template in PCR reactions using 18 primers: Lv 319, Pb10, B4, C9, Lv 472, Pod 1B, B6, Pb73, Lv 4α Pod 2, Po47, Po56, Po55, Po22, Po11, Po43, Po51 and Po18 [60,61]. PCR reactions were carried out in a GeneAmp PCR system 9700 (Applied Biosystems Inc., Foster City, CA, USA) and the conditions used are specified in [61] and [60] respectively. The PCR products were separated and alleles were detected in an ABI PRISM 3730 capillary sequencer (Applied Biosystems). GeneMapper (Applied Biosystems) was used to determine the genotypes of the individuals.

### Molecular population genetic analyses

We obtained genetic data from 289 individuals from the eight study populations (see Additional file 1 for breakdown of sample sizes for the different populations) and each of these individuals was scored at the 18 microsatellite loci. Departure from Hardy-Weinberg expectations and the genetic differentiation (F_ST_) between populations was estimated with FSTAT [62]. F_ST _for throat color morph was also calculated in FSTAT; the three colors were coded as alternative alleles at one locus. This analysis thus assumes that throat color is a heritable trait with Mendelian inheritance and codominant expression. We assumed that single color and combinations of two colors correspond to homo- and heterozygous stages, respectively. The program Structure version 2.2 [63] was run on individual multilocus genotypes for a number of clusters *K *ranging from 1 to 12 using a burn-in length of 50,000 and a run length of 100,000 iterations. The likelihood for the data given each of *K *clusters was recorded, but since the main aim of the analysis was to illustrate the degree of genetic differentiation between populations rather than to find the number of genetic clusters for which the likelihood is highest, the additional method [64] for determining *K *was not applied. Effective population size N_e _was estimated with LDNE [65]. AMOVAs were performed in Arlequin version 3.3 [66] to evaluate the within- and among group genetic variance. Isolation-by-distance was tested using Mantel's test implemented in the ISOLDE application in the program GENEPOP [67]. We used a general linear model (GLM) with color morph F_ST _as dependent variable and neutral F_ST _and category (mainland-mainland, mainland-island and island-island) as main effects and used a design including all the two-way combinations as well as the three-way combination, to test both for dependence on the two main effects and interactions between them. Since each population is involved in more than one comparison the data points are not independent, so we used a resampling approach [68] to confirm these results. We also used regressions to test for a correlation within the individual categories mentioned above, and used resampling statistics [68] to confirm our results since the data points are interdependent. The resampling procedure confirmed the results from the GLM, and hence we only present the results from the latter in this study. We thus compared the F_ST_-values calculated from the color morphs ("morph divergence") and the F_ST_-values based on the 18 microsatellite loci ("neutral divergence").

Selection can be inferred from the genetic signature of different molecular markers; markers that have been under directional selection are expected to be more diverged than neutral markers and markers that have been subjected to stabilizing selection should be less diverged [44,45]. Recently, it has been argued that comparisons of population divergence between putatively adaptive traits (often referred to as "Q_ST_") and neutral population divergence (F_ST_), should utilize the whole neutral F_ST_-distribution to see if the adaptive traits fall within this distribution, before the null hypothesis of genetic drift can be safely rejected [16,44]. We follow these recommendations and visualized the entire neutral F_ST_-distribution and its relationship to our adaptive trait (color morph divergence). We used a paired t-test to investigate if F_ST _for throat color morph differed from the F_ST _for the microsatellite loci. This procedure assumes that the pair-wise population comparisons are statistically independent, which is questionable, since each population is involved in several different comparisons. We therefore confirmed the results from the paired t-test using the re-sampling procedure described above [68], but our conclusions remained the same (see Results). To investigate if the morph frequencies on the islets have evolved in parallel, we performed a logistic regression with a binomial distribution and a logit link, where orange allele or not orange allele is the dependent variable and habitat (islet or mainland) the main effect. The subspecies *P. g. weigandi *was not included in this analysis since the habitat of Piperi is expected to differ from that of Skyros and its' surrounding islets and thus not reflect an islet- or mainland habitat effect.

## Results

### Color morphs: visual classification and spectral analysis

**Figure 3:**
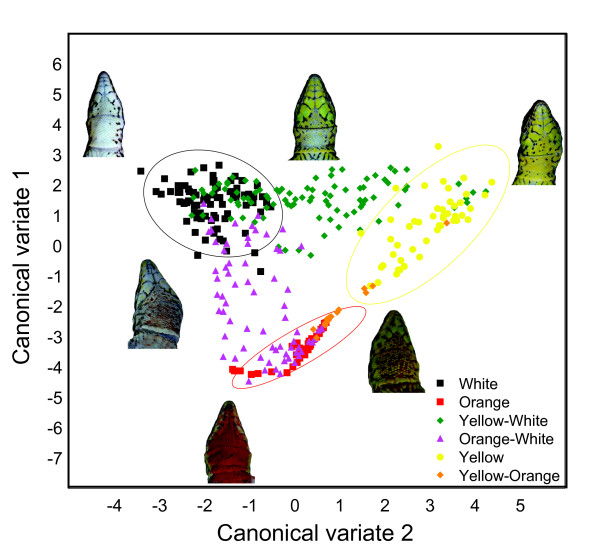
**Discreteness of throat color morphs**. Canonical roots (CV) of throat color morphs. Lizards were classified to one out of six possible morphs visually (symbols) and the canonical roots (CV1 and CV2) were calculated from the spectral information that we obtained from RGB photographs. Shown are 95% confidence ellipses for the three "pure" or putatively 'homozygous' throat color morphs (O, W and Y).

A discriminant function analysis based on the seven first principle components extracted from the throat images revealed that the six visually classified throat color morph groups were highly statistically different from each other (*F *= 31.81, df = 7, 318, *P *< 0.001) (Fig. 3). 14 out of 15 post-hoc tests between the different groups were highly significant (*P *< 0.001), with the only exception for the yellow-orange morph that did not differ from the orange (*P *= 0.11). The results from these analyses, showing variation from the first two canonical roots is depicted in Fig. 3. Of the six phenotypes that were clearly separated from each other, the confidence ellipses of the three "pure" morphs (putative homozygotes) are also shown (Fig. 3).

### Geographic variation in color morph frequencies

**Figure 4:**
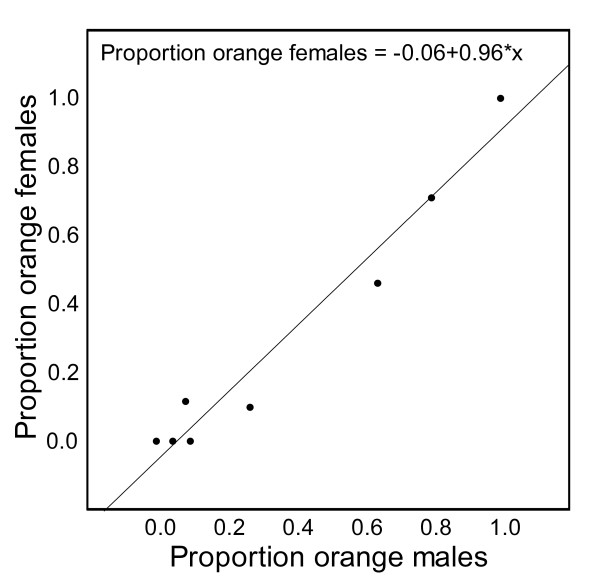
**Correlation between male and female throat color**. Positive relationship between the local proportions of orange females and orange males in each of the eight study populations. The regression line *y *= 0.06 + 0.96*x is statistically significant (see text).

Color morph frequencies differed significantly between all eight populations (χ² = 1168, df = 35, *P *< 0.001). The frequencies also differed significantly between the mainland group and islet group within the subspecies *P. g. gaigeae *(χ² = 336, df = 5, *P *< 0.001) (Fig. 2B). All main colors (O, Y, W) - although not all six throat color morphs - were found in all mainland populations and in two of the three islet populations of *P. g. gaigeae *(Fig. 2B). On the third islet, only 16 individuals were sampled, and we only found Y and W. In the other subspecies (*P. g. weigandi*), only O and W were found (N = 31). We found a significant positive relationship (β = 0.88; *P *< 0.05) between the proportion of females in the populations with partly or fully orange throats (e. g. O, YO, OW individuals) and the proportion of males with partly or fully orange throats (Fig. 4). This suggests that the color polymorphism in this species is present in both males and females and that it is not sex-limited in its expression. None of the eight populations differed significantly from Hardy-Weinberg expectations with respect to local throat color morph frequencies (Additional file 2).

### Genetic population differentiation

Pair-wise population comparisons of neutral genetic variation (F_ST_-values between populations) ranged from 0.0057 to 0.52 when *P. g. weigandi *was included and between 0.0057 and 0.36 within *P. g. gaigeae *only (Table 1). All between-population comparisons of neutral divergence were highly significant (*P *< 0.001). F_ST_-values estimated from the color morph frequency data ranged from 0.0025 to 0.45 when all populations and *P. g. weigandi *were included, and between 0.0025 and 0.24 within *P. g. gaigeae *only (Table 1).

**Table 1:** F_ST _for neutral genetic variation and throat color morph

Population	Agios Fokas	Atsitsa	Mesa Diavates	Exo Diavates	Lakonissi	Nyfi	Palomares	Piperi
Agios Fokas (M)		0.0165	0.1530	0.1002	0.2249	0.0261	0.0286	0.2941
Atsitsa (M)	0.0025		0.1908	0.1096	0.2587	0.0486	0.0057	0.3399
Mesa Diavates (I)	0.0727	0.0821		0.2196	0.3641	0.1486	0.1970	0.4206
Exo Diavates (I)	0.0850	0.1655	0.2030		0.3300	0.0726	0.1287	0.3632
Lakonissi (I)	0.1990	0.2404	0.0830	0.2205		0.2515	0.2561	0.5200
Nyfi (M)	0.1178	0.1930	0.2353	0.0088	0.2417		0.0488	0.2729
Palomares (M)	0.0234	0.0031	0.0493	0.2181	0.2191	0.2452		0.3365
Piperi (I)	0.3376	0.3859	0.1383	0.4501	0.0711	0.4510	0.3333	

F_ST_-values based on 18 microsatellite loci (upper right half) and F_ST_-values based on throat color phenotype frequency in the population (lower left half). Mainland localities are abbreviated with a M and island locals with an I.

**Figure 5:**
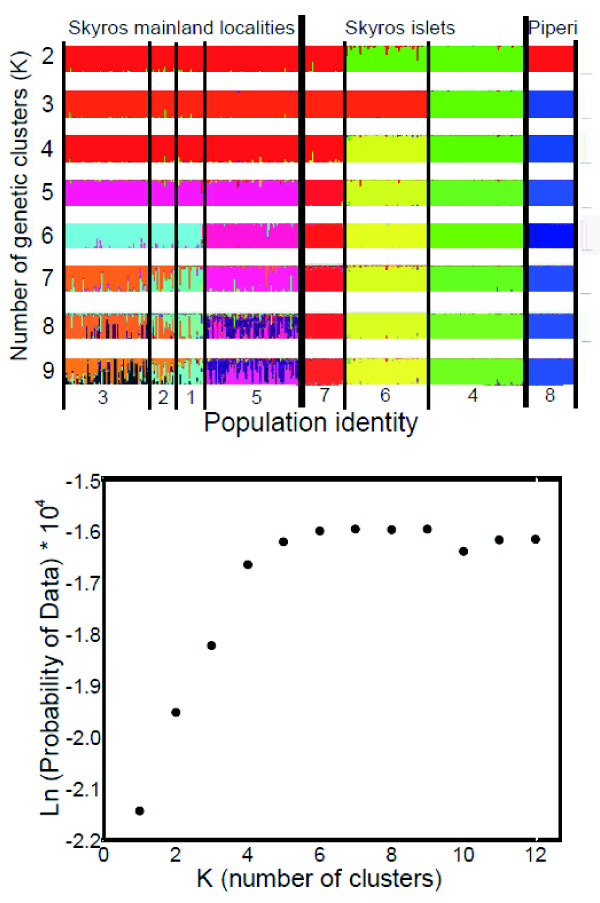
**Genetical clustering of populations**. (A) Genetical clustering when the eight sampled populations of *P. gaigeae *are grouped into *K *= 2-9 clusters using the STRUCTURE algorithm [63]. (B) Log-likelihood of the microsatellite data given *K *clusters.

We used the microsatellite data and STRUCTURE [63] to illustrate the degree of genetic population structure and to infer the number of genetically independent populations (or "clusters"; *K*) that we could find support for in our data set (Fig. 5). The likelihood of data LnP(D) stabilized within 20,000 iterations for all tested values of *K*, and thus a run length of 100,000 was deemed as sufficient. The clusters defined by STRUCTURE correspond to a sampling locality or a group of sampling localities up to *K *= 6 (Fig. 5A) and the log-likelihood-values reached a plateau at *K *= 6 (Fig. 5B). At *K *= 7 all sampling localities were well defined, with the exception of the two most northern mainland populations that included a majority of individuals assigned to two different genetic clusters (populations 1 and 2 on the map). At *K *= 2, two of the island populations form a separate cluster and at *K *= 3 the *P. g weigandi *population form a third cluster. The next two clusters to form are the remaining island populations, and at *K *= 6 the southern mainland population separates from the three northern mainland populations (Fig. 5 A).

Estimates of effective population sizes (N_e_) based on all allele frequencies > 0.01 varied between 39.4 and 97.7 for the islet populations of *P. g. gaigeae *and between 315.3 and 375.0 for the mainland (Skyros) populations (Table 2). The N_e _of *P. g. weigandi *and two of the northern mainland populations of *P. g. gaigeae *could not be accurately estimated due to either a too high noise-to-signal ratio or a very large population size. Using 0.02 or 0.05 as lowest frequency of alleles included in the analysis yielded similar results (data not shown).

**Table 2:** Effective population sizes

Population	N_e_	Lower 95%CI	Upper 95%CI
Mainland 1 (AF)	315.3	202.7	669.9
Mainland 3 (AT)	-	-	∞
Island 2 (D)	97.7	66.3	170.0
Island 3 (DII)	39.6	27.1	66.7
Island 1 (L)	39.4	29.7	54.6
Mainland 2 (N)	375.0	240.8	802.9
Mainland 4 (P)	-	177.0	∞
*P. g. weigandi*	-	288.2	∞

Estimated effective population size (N_e_) for the studied populations. Alleles with frequencies above 0.01 were included in the analysis.

Most of the genetic variance was found within populations (more than 75%; Table 3). The variance was low among groups (2.6%) but higher among populations (14.9%) when the populations were divided into an island and a mainland group (Table 3). When the mainland populations were grouped in two populations (north and south), both the between-group (1.6%) and the among-population variance (2.3%) were low (Table 3). When the islets were divided into two groups (northern and southern), the between-group variance was still rather low (6.8%), but among-population variance (23.3%) was nevertheless an order of magnitude higher than the among-population variance when only the mainland populations were included in the analysis (Table 3). This pattern of within- and among-population variation is consistent with a scenario where the islet populations have lost different components of the genetic variance, presumably a result of their lower effective population sizes (Table 2). The mainland populations of *P. g. gaigeae *showed a pattern of differentiation consistent with isolation-by-distance (Mantel's test; *y *= -0.27 + 0.033×ln(distance), *P *= 0.04). There was no such trend when the islet populations of *P. g. gaigeae*, or when *P. g. weigandi *and the islet populations, were included in the analysis, however (Table 4).

**Table 3:** Partitioning of genetic variance

Grouping	Among groups	Among populations	Within populations
Mainland (1, 2, 3, 5), Islets (4, 6, 7), *P. g. W *(8)	10.31	13.91	75.78
Mainland (1, 2, 3, 5), Islets (4, 6, 7)	2.61	14.89	82.50
North mainland (1, 2, 3), South mainland (5)	1.63	2.37	96.0
North islet (4), South islets (6, 7)	6.82	23.32	69.86

AMOVA for genetic variance for different groupings of the studied populations. Variance is partitioned into among group variation, among population variation and within population variation.

**Table 4:** Isolation by distance

Group	a	b	*P*
*P. g. gaigeae *and *P. g. weigandi*	-0.98	0.13	0.11
*P. g. gaigeae*	0.53	-0.04	0.65
*P. g. gaigeae *mainland	-0.27	0.03	0.04

Isolation by distance, where population differentiation (F_ST_) = a + b*ln(geographical distance (km)). *P*-values are from Mantel's tests.

**Figure 6:**
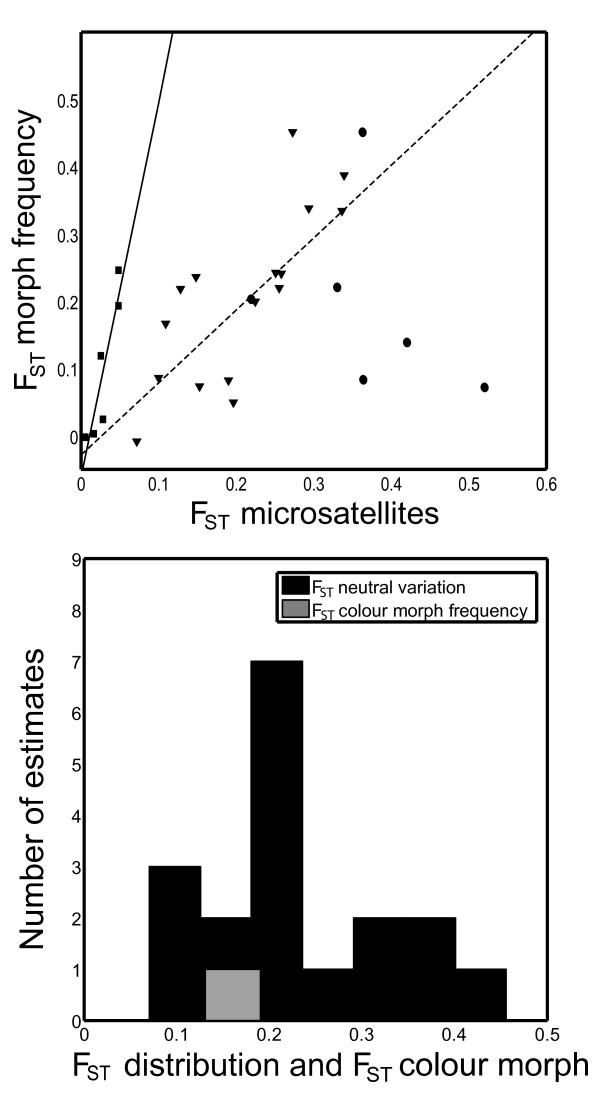
**Relationship between neutral divergence and morph divergence**. (A) Relationship between the pair wise population comparisons (F_ST_) calculated from color morph frequencies (assuming a single locus character) and neutral genetic divergence (F_ST _obtained from 18 microsatellite loci). The relationship is positive and statistically significant when data from all categories are included: *y *= 0.09 + 0.44*x; *r*^2 ^= 0.20; *F *= 6.37; df = 1, 26; *P *= 0.02. There is also a significant relationship when only the mainland populations are included (squares; full line; *y *= -0.07 + 5.59*x; *r*^2 ^= 0.83; *F *= 19.41; df = 1, 4; *P *= 0.01) and when only mainland-island comparisons are included (triangles; dashed line; *y *= -0.04 + 1.19*x; *r*^2 ^= 0.60; *F *= 21.01; df = 1, 14; *P *= 0.0004). There is no significant relationship between color morph divergence and neutral divergence when only island populations are compared (dots). (B) Distribution of mean pair wise F_ST_-values for the 18 microsatellite loci (black) and the mean pair wise F_ST_-values that were calculated from the throat color morph frequencies (assuming a single locus character; (in white)).

Color morph divergence was significantly correlated with neutral genetic divergence across all populations (*F *= 6.37; df = 1, 26; *P *= 0.02). When we separated these comparisons into three different geographic categories (mainland-mainland, mainland-island and island-island), we found a significant positive relationship within two of these categories (Fig. 6A). The mainland-mainland and mainland-island comparisons were both positive and significant, whereas the island-island comparison was non-significant (Fig. 6A). The interaction term between geographic category and neutral divergence was significant (*F *= 7.5; df = 4, 48; *P *= 0.003), indicating that the correlation between color morph divergence and neutral divergence differed between the geographic settings (Fig. 6A).

Mean F_ST_-values of color morph frequencies clearly fell within the range of the mean F_ST_-values based on each one of the 18 microsatellite loci (Fig. 6B). No difference was found between F_ST _based on color morph frequencies and F_ST _based on neutral genetic divergence (Paired t-test: *t *= 0.91; df = 27; *P *= 0.37; results remained the same after resampling). Hence these data do not allow us to firmly exclude that population divergence in color morph frequencies have been affected solely by genetic drift. This conclusion remains the same if the categories are analyzed separately (all *P*-values > 0.05). Islet populations had a significantly higher proportion of orange throat color alleles than mainland populations (logistic regression: χ^2 ^= 112.91; df = 1; *P *< 0.001), although no orange throat color alleles were found on one of the three islets.

## Discussion

What is the role of genetic drift in phenotypic evolution, if any? Even if most evolutionary biologists have strong reasons to believe that genetic drift is a weak force in phenotypic evolution, in comparison to selection [69,70], genetic drift can still influence trait variation that later becomes visible to selection [23]. For example, the loss of a particular color morph might change the social selective environment, particularly when color is used in intra- or intersexual signaling [46,71]. Genetic drift in sexually selected traits in isolated populations might lead to profound consequences upon secondary contact. Since sexually selected characters can diverge along a neutral line of equilibrium [72], genetic drift in sexually selected characters can result in sexual isolation between populations as a by-product of sexual selection within populations [11]. An interaction between sexual selection and genetic drift is of particular interest in the present study as well as in other lizard species, since throat color in lizards has been found to be correlated with immunological condition and color could thus function as a sexually selected honest signal both within and between the sexes [73].

Comparisons of phenotypes from island and mainland populations played a central role in Ernst Mayr's theory of founder effect speciation [1,74] and continues to inspire contemporary evolutionary biologists today [75]. For instance, island biology studies of enigmatic color morphs in Dendrobates frogs have shown an impressive color morph diversity in the archipelago of Bocas del Toro, outside Panama, compared to mainland Central America [76]. The discrete throat color phenotypes in *P. gaigeae *and the natural geographical replicates (i.e., different islet populations), as well as the variation in throat color throughout the Balkan clade of *Podarcis *where the sister species *P. milensis *does not exhibit throat color morphs whereas for example *P. melisellensis *has retained it, make this an excellent study system to address how selective processes operate in subdivided islet populations.

A role for phenotypic plasticity in determining the throat color morphs in *P. gaigeae *can not be excluded. If throat color morph would be entirely plastic with no heritable basis, the significant correlations between neutral genetic variation and the throat color morph frequencies for mainland-mainland and islet-mainland population pairs (Fig. 6A) would still need an explanation. One possibility might be that these correlations could reflect habitat differences between populations that have diverged to different degrees and at a rate that is proportional to molecular divergence. One could possibly argue for this possibility on the main island if there would be a clinal change in habitat. However, such a correlation would not necessarily be expected between the islet-mainland population pairs since the similarity in habitat between the islets and mainland locals is unlikely to be proportional to the time since the islets were isolated from the mainland. Rather, the environments on the islets depend more on local factors such as soil type and anthropogenic impact such as grazing pressure from goats (A. Runemark and E. Svensson, unpublished observations). Although we do not exclude the existence of genotype-by-environment interactions, a scenario with no heritable basis at all for the throat color polymorphism seems quite unlikely. With the caveat that we have no data from breeding experiments on the genetic basis of this throat color polymorphism, the discrete nature of these color morphs (Fig. 3), the strong correlation in throat color between the sexes (Fig. 4), our findings that none of the populations deviate from Hardy-Weinberg proportions with respect to the throat color morphs (Additional file 2), and the significant relationship between population divergence of the throat color morphs and neutral genetic divergence (Fig. 6A), is consistent with a heritable basis of throat color. Previous studies on several other lizard species have often found indications of a relatively simple genetic architecture of color morphs [30-34]. Although we do not know the exact number of loci involved in determining the throat color, the results are based on allele frequencies, and even if more than one locus would determine the throat color, it would not qualitatively change our conclusions, as long as there is some genetic basis to the polymorphism (e. g. a major-effect-gene). Based on the evidence above we tentatively assumed a simple genetic basis, and investigated if the data on the spatial distribution of these color morphs is consistent with the neutral expectation, and that stochastic factors alone might responsible for the population differentiation.

In the genus *Podarcis *several, if not most, species exhibit throat color polymorphism which indicates that these polymorphisms might have survived several lineage splitting and speciation events [48], (Fig. 1). When a polymorphism has been retained from a common ancestor it is highly unlikely to be entirely selectively neutral because strong overdominance or negative frequency-dependent selection is required to maintain all morphs in such systems [77-79], and selection on traits, including color polymorphisms, is usually a strong force [14]. Thus, it is probable that there is long-term balancing selection that maintains the throat color morphs in *P. gaigeae *and other *Podarcis*-species. In addition, if throat color morph was selectively neutral it would be highly unlikely that all three alleles would be present on all mainland locations and two out of three islets. Consistent with such a scenario where selection acts in a stabilizing fashion, the frequencies of the throat color morphs were quite similar across all the mainland populations on Skyros (Fig. 2B). Interestingly, the orange throat color morphs were overrepresented on the islets compared to the mainland populations (Fig. 2B). These differences between mainland and islet populations could potentially be a result of different local selective environments on the islets or islet specific genotype by environment interactions if such exist, although we have no direct ecological data in support of this. Subdivided populations with limited effective population size are, however, also affected by genetic drift [80]. Thus, the differences in throat color morph frequencies between the geographically close islet populations with similar habitats (Figs. 2A-B) could potentially partly be a result of stochastic processes.

Random fluctuations due to genetic drift can result in a form of environmental noise that can in turn fuel selection. For instance, when negative frequency-dependent selection operates in a system with environmental noise, perturbations in one direction can generate strong backlashes, which in turn can fuel selection and cause rapid evolutionary dynamics [25]. When the selective signal is weak and environmental noise is high, stochastic factors might partly and temporarily overcome the selective signal (see for example [80]). Stochastic effects such as genetic drift have a larger impact when population size is small [17], as each stochastic death implies a larger change in the allele frequency distribution. Moreover, when one of the alleles is maintained at a low frequency at stable evolutionary equilibrium, selection may accelerate the loss of the rarest allele by random genetic drift [17]. In several other color polymorphic systems, including species with continuous distributions and large population sizes, the frequency of the different color morphs sometimes shows patterns which are not distinguishable from the expectations under a scenario of genetic drift [7,9,23]. In addition to genetic drift, other forms of stochasticity can also operate and affect allele frequencies, even in large populations [12]. These forms of stochasticity include demographic stochasticity and stochasticity generated by life-history variation among genotypes [12].

Three different lines of evidence suggest that genetic drift might partly be responsible for the divergence in throat color morph frequencies between the islets given that the throat color polymorphism is heritable (Fig. 2B). First, the effective population sizes on the islets are very low (Table 2; All N_e_:s < 100). In such small populations, stochastic frequency fluctuations in selected alleles might under some periods temporarily override the effects of selection [80]. With an effective population size of approximately 40 individuals, as is the case for one of the islets, there are only 80 throat color alleles present and random loss of only a few individuals could potentially have large effects on the overall population allele frequency. Second, the F_ST_-values of traits subjected to directional selection are expected to differ significantly from the F_ST_-values for neutral genetic variation [44,45]. The F_ST _for throat color morph frequency fell in the lower range of the distribution of the F_ST_-values for neutral genetic variation (Fig. 6B). Comparisons of population divergence for different sets of loci are useful for identifying loci under directional selection [44], although the large confidence intervals which sometimes overlap zero can make it difficult to infer stabilizing selection with this approach [81]. The overall high neutral F_ST_-value for this lizard species (0.208) should, however, increase our statistical power to detect stabilizing selection in this system that might have favored a certain stable morph frequency equilibrium in all populations. Third, we found a significant positive correlation between the population differentiation in throat color and neutral genetic divergence (Fig. 6A). If selection would mainly explain the population morph frequency differences, we would not necessarily expect a change in genome-wide neutral genetic variation. Selection pressures to maintain a stable morph frequency across all populations might be the appropriate null expectation of population divergence for polymorphisms that transcend species boundaries, and when alleles are maintained by negative frequency-dependent selection [77-79]. If this is the case also for a trait like this color polymorphism, we would expect a pattern of population divergence for color morphs that would be significantly less than the neutral expectation at evolutionary equilibrium [15].

We do certainly not claim, based on these results, that genetic drift is the *only *evolutionary force operating on this color polymorphism. An additional role for selection and possibly for phenotypic plasticity can certainly not be excluded. Although indirect inferences about the action of selection are useful and have been used successfully by many workers in the past [15,82-84], such indirect approaches suffer from several limitations, among them low statistical power [45,81]. Failure to reject the drift-expectation for color morph divergence in this study (Fig. 6B) does thus not justify any strong claim that selection does not operate at all on these color morphs. Rather, we suggest that selection on these morphs, at least on the small islets, is not strong enough to result in a significant adaptive signal when we compared adaptive divergence and neutral divergence. Although genetic drift is usually considered by many population geneticists to be a weak force compared to selection [69,70], we tentatively suggest that an interaction between genetic drift and local natural or sexual selection is most likely explanation for the spatial patterns in our data. Genetic drift could change trait frequencies when selection is temporally relaxed, an ecological scenario that is likely to be quite common and which might exist in this system, for instance, when lizard populations invade novel island environments with fewer predators. We can not disentangle the relative contributions of genetic drift and founder effects based on our findings in this study. Since the sea level in the Aegean Sea has been rising and sinking [52,53] a scenario where lizards populations were isolated when land bridges were submerged and subsequently lost genetic variation due to low effective population sizes is probably more plausible than a pure founder event scenario, though.

The physical and biotic environments differ substantially among these small islets, which could generate novel selection pressures compared to the mainland. For instance, the predator faunas (birds and snakes) are more depauperate on the islets, and the seabird colonies on the islets might alter the nutrient cycles considerably, which in turn might affect the quantity and quality of the food sources of the lizards [50]. It is possible that the islet populations have adapted locally to meet the requirements of these island specific environments, or that genotype-by-environment-interactions result in certain colour morphs becoming expressed more often in certain environments. The vegetation on the islands in the Skyros archipelago differs substantially even among nearby islets, which might partly reflect stochastic factors and reveal some degree of community drift [75]. For instance, some common plant taxa are lacking entirely on some of the islets, whereas the abundance of other plant taxa is much higher on some of the islets than on the mainland [55]. Although these different habitat features might potentially favor different throat color morphs on different islets, the mainland populations also differ in many environmental variables, such as type of substrate, grazing pressure, vegetation cover and predator faunas (A. Runemark & E. I. Svensson, unpublished observations).

After the geographic isolation of *P. g. weigandi *from *P. g. gaigeae*, the orange morph increased in frequency on Piperi (Figs. 2A-B), perhaps as a result of founder events, genetic drift, or because of local selection favoring that morph in this novel island environment. If the different throat color phenotypes are genetically correlated to other physiological, morphological or behavioral traits, which is the case in *U. stansburiana *[26,28,32,85] or in immune response as is the case for *P. muralis *[27], one or several of the throat color morphs in the ancestral population could initially and due to chance alone, have been better adapted to the novel environments. Such "pre-adapted" morphs might then subsequently rise in frequency through phenotype sorting [86,87], either by selection alone or due to an interaction between selection and genetic drift. The loss of one or more color morphs has occurred several times in different populations of *U. stansburiana *[46,71], and it might also operate in the Balkan clade of *Podarcis *(see e. g. *P. milensis *in Fig. 1; [48]).

If genetic drift can affect phenotypic divergence in characters such as throat color which are often genetically correlated with other traits (see discussion above), genetic drift could potentially indirectly also change the optimal strategies for the remaining morphs since the selective environment will change if one morph and its strategy is removed. For instance, populations of *U. stansburiana*, which have lost morphs and become monomorphic, differ from the polymorphic populations with respect to sexual size dimorphism and potentially sexual selection [70]. Thus, the loss of one or several morphs might cause rapid divergence following a change in social selective environment in lizards. Polymorphic types within a population can thus potentially serve as starting material for new species [47,88,89] and the potential loss of the yellow throat color morph in the subspecies *P. g. weigandi *is interesting from this point of view.

## Conclusions

The color morph frequency in *P. gaigeae *differs among populations, with more pronounced frequency differences among islet populations. This might indicate a role for local genetic drift on color morph frequency divergence, although it is likely that these color morphs are maintained by long-term balancing selection, given the trans-species nature of these polymorphisms. Comparisons of neutral and morph frequency divergence did not reveal any evidence for either directional or stabilizing selection, which might partly be explained by the low statistical power and weak selection, rather than color morphs being entirely neutral. We therefore suggest that an interaction between selection and genetic drift explains the patterns of divergence in throat color morphs. In isolated populations with small effective population size, genetic drift is particularly likely to operate and to interact with selection which might cause divergence even in phenotypic characters.

## Authors' contributions

AR participated in the design of the study, carried out the field work, the color measurements and spectral analysis, the genetic analysis and drafted the manuscript. BH planned the genetic labwork and helped out with the design and conduction of the genetic analysis. PP and EDV helped out with the field work. EIS conceived of the study, and participated in its design and and helped to draft the manuscript. All authors read and approved the final manuscript.

## Supplementary Material

Additional file 1 **Number of photographed males and females per population**. Contains breakdown sample sizes for number of individuals per population and sex for which throat color morph was determined. Click here for file

Additional file 2 **Tests for deviation from Hardy-Weinberg equilibrium per population**. Contains tests for deviations from Hardy-Weinberg equilibrium for all populations and the breakdown sample sizes for number of genotyped individuals per population. Click here for file
