# Supplementary material for: Island biology and morphological divergence of the Skyros wall lizard Podarcis gaigeae: a combined role for local selection and genetic drift on color morph frequency divergence?
Source: BMC Evol Biol. 2010 Sep 2;10:269. doi: 10.1186/1471-2148-10-269 (PMC2939580; doi:10.1186/1471-2148-10-269)
Supplement: Additional file 1 — Number of photographed males and females per population. Contains breakdown sample sizes for number of individuals per population and sex for which throat color morph was determined. [file 1471-2148-10-269-S1.PDF]

Additional file1. Number of photographed males and females per population

Table 1. Number of individuals of *P. gaigeae* for which throat color morphs were determined divided per population and sex. Populations caught on localities on the main island of Skyros are denoted “mainland” and populations on islets close to the coast of Skyros and the population of the subspecies *P. g. weigandi* are denoted “islets”. Numbers after the population names correspond to the numbers of the localities on the map in Fig. 2.

**Habitat**	**Population name**	**Number of males**	**Number of females**
Mainland	Agios Fokas (3)	36	33
Mainland	Nyfi (5)	42	32
Mainland	Atsitsa (2)	11	9
Mainland	Palamari (1)	11	10
Islets	Lakonissi (4)	40	22
Islets	Mesa Diavates (6)	29	28
Islets	Exo Diavates (7)	11	5
Islets	Piperi (8)	14	17
