# Supplementary material for: Island biology and morphological divergence of the Skyros wall lizard Podarcis gaigeae: a combined role for local selection and genetic drift on color morph frequency divergence?
Source: BMC Evol Biol. 2010 Sep 2;10:269. doi: 10.1186/1471-2148-10-269 (PMC2939580; doi:10.1186/1471-2148-10-269)
Supplement: Additional file 2 — Tests for deviation from Hardy-Weinberg equilibrium per population. Contains tests for deviations from Hardy-Weinberg equilibrium for all populations and the breakdown sample sizes for number of genotyped individuals per population. [file 1471-2148-10-269-S2.PDF]

Additional file 2. Tests for deviation from Hardy-Weinberg equilibrium per population

Table 1. $\chi 2$-test for deviation from Hardy-Weinberg frequencies (HW) in throat colour morph within populations assuming a one locus three allele codominant inheritance. Degrees of freedom (df) was calculated as number of possible genotypes – number of alleles. None of the populations deviated significantly from the expected distribution, and the null hypothesis that the populations are in Hardy-Weinberg equilibrium with respect to the colour morph can thus not be rejected.

**Population**	**$\chi 2$**	**df**	**Null hypothesis of HW rejected at 5% level**	**Number of sampled individuals**
Palamari (1)	0.32	3	No	21
Atsitsa (2)	0.23	3	No	20
Agios Fokas (3)	0.27	3	No	69
Lakonissi (4)	0.50	3	No	62
Nyfi (5)	0.43	3	No	74
Exo Diavates (6)	0.56	3	No	17
Mesa Diavates (7)	0.38	1	No	57
Piperi (8)	0.42	1	No	31
**Goodness of fit**	**3.1**	**3**	**No**	**289**
